# Biomimetic Mineralization of Tooth Enamel Using Nanocrystalline Hydroxyapatite under Various Dental Surface Pretreatment Conditions

**DOI:** 10.3390/biomimetics7030111

**Published:** 2022-08-11

**Authors:** Pavel Seredin, Dmitry Goloshchapov, Vladimir Kashkarov, Anna Emelyanova, Nikita Buylov, Konstantin Barkov, Yuri Ippolitov, Tatiana Khmelevskaia, Iman A. Mahdy, Manal A. Mahdy, Tatiana Prutskij

**Affiliations:** 1 Solid State Physics and Nanostructures Department, Voronezh State University, University Sq. 1, 394018 Voronezh, Russia; 2 Scientific and Educational Center “Nanomaterials and Nanotechnologies”, Ural Federal University, Mir Av., 620002 Yekaterinburg, Russia; 3 Department of Pediatric Dentistry with Orthodontia, Voronezh State Medical University, Studentcheskaya St. 11, 394006 Voronezh, Russia; 4 Physics Department, Faculty of Science (Girls), Al-Azhar University, Nasr City 11754, Cairo, Egypt; 5 Solid State Physics Department, National Research Centre, Dokki 12622, Giza, Egypt; 6 Sciences Institute, Autonomous University of Puebla (BUAP), Puebla 72570, Mexico

**Keywords:** biomimetic, mineralization, enamel tissue, FESEM, AFM, XRD, Raman spectromicroscopy, nanoindentation

## Abstract

In this report, we demonstrated the formation of a biomimetic mineralizing layer obtained on the surface of dental enamel (biotemplate) using bioinspired nanocrystalline carbonate-substituted calcium hydroxyapatite (ncHAp), whose physical and chemical properties are closest to the natural apatite dental matrix, together with a complex of polyfunctional organic and polar amino acids. Using a set of structural, spectroscopy, and advanced microscopy techniques, we confirmed the formation of a nanosized ncHAp-based mineralized layer, as well as studying its chemical, substructural, and morphological features by means of various methods for the pretreatment of dental enamel. The pretreatment of a biotemplate in an alkaline solution of Ca(OH)_2_ and an amino acid booster, together with the executed subsequent mineralization with ncHAp, led to the formation of a mineralized layer with homogeneous micromorphology and the preferential orientation of the ncHAp nanocrystals. It was shown that the homogeneous crystallization of hydroxyapatite on the biotemplate surface and binding of individual nanocrystals and agglomerates into a single complex by an amino acid booster resulted in an increase (~15%) in the nanohardness value in the enamel rods area, compared to that of healthy natural enamel. Obtaining a similar hierarchy and cleavage characteristics as natural enamel in the mineralized layer, taking into account the micromorphological features of dental tissue, is an urgent problem for future research.

## 1. Introduction

Recently, a great deal of effort has been made to study the mechanisms and effective approaches for the directed remineralization of dental enamel [1,2,3,4]. This is due to the fact that the regeneration of enamel, which is a highly mineralized hard tissue with a multilevel hierarchical structure, is a difficult task [1,5,6]. To date, using a variety of biomedical strategies of all kinds, no laboratory-appropriate ways of cloning an enamel-like structure for medical engineering applications have been obtained [2,7,8,9]. However, there is currently a belief in the scientific community that the damaged microstructure of natural enamel can be not only restored but also precisely copied [4,6,7,8]. A prerequisite for these beliefs was the development of the strategy of biomimetic mineralization, which is based on the study of biological processes during which living organisms create materials with unique local properties from nature, as well as on the use of natural technologies to create artificial bionic structural materials [4,8,10,11,12].

It is well known that human teeth are formed with the participation of specialized cells under the control of the genetic code during long-term natural mineralization [5,6]. Therefore, induced biomimetic mineralization must occur in a transient amorphous–crystalline interface layer at the enamel boundary, and a set of different amino acids is required to control its processes and form a biosimilar tissue [13,14,15,16,17]. Amino acids form an organic matrix similar to that of natural enamel [18], provide crystallization processes, and determine the orientation and binding of inorganic subunits in the formation of a highly organized enamel-like structure [13,14,17,19,20].

However, the biomimetic approach to the mineralization of dental tissue is not simply about “replacing” it but is simultaneously about studying the principles and mechanisms of its reconstitution [11,12,21]. Therefore, the fundamental problem and the key point in the ideal restoration of dental enamel is the search for an effective technology to duplicate the anisotropy, morphology, and optical and biomechanical properties [7,22,23,24] defined by the biotemplate (natural tissue).

Thus, it has already been demonstrated that the assembly of an apatite-like inorganic, oriented structure on the surface of natural enamel can be organized using calcium phosphate nanoparticles [7,8,25]. However, inducing the qualitative growth of hydroxyapatite (HAp) crystals commensurate with natural enamel apatite crystals using nanoclusters on the biotemplate has only been partially possible. To achieve the necessary crystal density, chemical resistance, mechanical strength, and hardness of the mineralized layers, a necessary condition is the interaction and conjugation of HAp crystals with the organic enamel matrix [22,23,24,26].

It is likely that the key to solving this problem is choosing the optimal approach to creating a biointerface between a natural and biomimetic material [2,27] and, in fact, a method of pretreatment of the natural tissue surface (biotemplate) for its subsequent effective mineralization [28,29]. Nanocrystalline carbonate-substituted calcium hydroxyapatite (ncHAp), which is the closest to being a natural dental matrix apatite in its physical and chemical properties, can serve as a suitable material [30,31,32]. However, an in-depth and quantitative analysis of the molecular structure of biointerfaces with a high (submicron) spatial resolution is required to solve this problem [33,34,35], which has also been an issue in research to date.

All these questions, in our opinion, have been given insufficient attention, and they remain open and topical, requiring a non-trivial resolution. Therefore, the aim of our work was to study the effect of the pretreatment of the tooth enamel surface on the formation of a biomimetic mineralized layer using ncHAp.

## 2. Methods of Production and Study of the Samples

### 2.1. Obtaining Enamel Samples

In our study, we used dental tissue samples taken from patients aged 20–25, to create biotemplates. Samples of the hard dental tissues conformed to the following criteria: completely sound enamel without carious lesions (ICDAS code 0–sound enamel without carious lesions) and without erosion or any other lesions (splits, cracks). Ten healthy human teeth were collected in compliance with the rules and regulations of the Declaration of Helsinki and with ethical protocol. The teeth were extracted from the patients for orthodontic reasons. The collected teeth were immediately mechanically cleaned of plaque, rinsed repeatedly with distilled water using a compressor, and placed in distilled water, where they were stored at +4 °C.

### 2.2. Sample Preparation

Dental segments were prepared for our studies. Using a low-speed (120 rpm) water-cooled diamond blade [36,37], the tooth samples were divided into segments. For this purpose, the blade was passed through the area of the fissure system in the direction perpendicular to the masticatory surface. The thickness of each segment was ~2 mm. An ultrasonic bath (transmitter power ~25 W) was used to remove residual contamination from the surface of the enamel segments. The treatment was performed in distilled water once for 60 s.

After the ultrasonic cleaning procedure, we selected 5 segments from each tooth, with a clean surface, without cracks or chips. A total of 50 segments were selected, which were further divided into 5 groups (*n* = 10 segments per group).

### 2.3. Sample Groups Treatment

**Figure 1:**
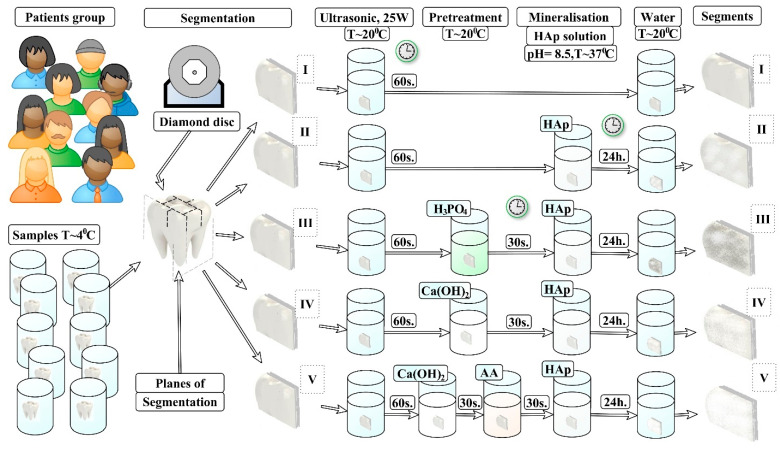
Scheme of ncHAp-based mineralized layer formation under different biotemplate pretreatment conditions. **Ⅰ**–**Ⅴ**: type of samples.

In accordance with the design of our experiment, each group of samples was subjected to the specified pretreatment procedure (see Figure 1).

#### 2.3.1. N1 Samples

The samples of this group were used as standards of healthy enamel (see Figure 1). 

#### 2.3.2. N2 Samples

No pretreatment of the surface of the samples was performed. The samples were stored in distilled water prior to the mineralization procedure (see Figure 1).

#### 2.3.3. N3 Samples

Pretreatment of the samples in this group was performed using the traditional-for-dental-practice procedure of etching the enamel. Etching was performed using a 38% solution of orthophosphoric acid H_3_PO_4_ for 30 seconds, to obtain a structured enamel surface. After the pretreatment procedure, the samples were washed and mineralized (see Figure 1).

#### 2.3.4. N4 Samples

Pretreatment of the enamel surface was performed by placing each sample in an alkaline solution of Ca(OH)_2_. The alkalizing time was 30 s. After the pretreatment procedure, the samples were washed and mineralized (see Figure 1).

#### 2.3.5. N5 Samples

Two pretreatment steps were performed for each of the samples in this group. In the first step, each sample in the group was placed for 30 s in an alkaline solution of Ca(OH)_2_. Next, each sample was washed with distilled water and placed for 30 s in an amino acid booster. The samples were then subjected to mineralization (see Figure 1).

### 2.4. Mineralization

The N2–N5 samples were placed in a freshly prepared solution that contained nanocrystalline carbonate-substituted hydroxyapatite. The pH value of the solution was 8.5. The mineralization procedure was performed for 24 h at room temperature. The enamel segments (samples) were then washed in distilled water and stored at 4 °C until the experiments were performed.

It should be noted that rather concentrated reactants were applied for the enamel pretreatment. H_3_PO_4_ acid is applied in clinical practice for enamel pretreatment. The standard time for this procedure is ~30 s. Therefore, in a similar way, we chose a time for treatment of the samples with the use of Ca(OH)_2_ equal to 30 s. A total of 24 h for the mineralization process was chosen, based on the analysis of a number of references where the time of mineralization varied within the limits of 5 to 96 h for different techniques [23]. We aimed to study the process of hydroxyapatite mineralization, and the most probable deposition of the layers was observed after 18–24 h.

### 2.5. Experimental Design

The design of the experiment is shown schematically in Figure 1.

### 2.6. Materials Used

#### 2.6.1. Ca(OH)_2_ Solution

To obtain the calcium hydroxide solution, we used bird eggshells as an initial reagent. Calcium carbonate contained in eggshells has a similar composition of micronutrients to that characteristic of mineralized (dental and bone) human tissues [38]. This fact is an advantage during the subsequent synthesis of bioinspired carbonate-substituted calcium hydroxyapatite (HAp), the physicochemical properties of which are closest to those of the natural apatite of a dental matrix [31].

The process of obtaining calcium hydroxide involved the high-temperature annealing of bird eggshells (2 h at 950 °C), during which the organic component of the shell burns and a highly active calcium oxide remains. A solution of Ca(OH)_2_ was formed by dissolving calcium oxide in distilled water. The concentrated alkali obtained was further diluted with distilled water to obtain a solution with pH = 12 and was used to treat the resulting tooth enamel segments. To prevent agglomeration, the alkaline solution was treated with ultrasound for 30 s. A titanium immersion probe and a Qsonica ultrasonic homogenizer (Qsonica Llc, Newtown, CT, USA) were used, with a transmitter power of 55W and an amplitude of 50%.

#### 2.6.2. NcHAp Solution

An aqueous solution of highly dispersed nanosized carbonate-substituted calcium hydroxyapatite (ncHAp) was obtained by chemical precipitation. For this purpose, calcium hydroxide obtained by the method described above was used.

We used bird eggshells as an initial reagent. Calcium carbonate contained in eggshells has a similar composition of micronutrients to that characteristic of mineralized (dental and bone) human tissues [38]. This fact is an advantage during the subsequent synthesis of bioinspired carbonate-substituted calcium hydroxyapatite (Hap), the physicochemical properties of which are closest to the natural apatite of a dental matrix [31].

The process of obtaining calcium hydroxide involved the high-temperature annealing of bird eggshells (2 h at 950 °C), during which the organic component of the shell burns and a highly active calcium oxide remains. A solution of Ca(OH)_2_ was formed by dissolving calcium oxide in distilled water. The obtained concentrated alkali was further diluted with distilled water to obtain a solution with pH = 12 and was used to treat the resulting tooth enamel segments. To prevent agglomeration, the alkaline solution was treated with ultrasound for 30 s. A titanium immersion probe and a Qsonica ultrasonic homogenizer (LLC, Newtown, CT, USA) were used, with a transmitter power of 55W and an amplitude of 50%.

Calcium hydroxide was titrated with a 0.3 M orthophosphoric acid (H_3_PO_4_) solution to a fixed pH (pH = 8.5) at room temperature under atmospheric conditions. Immediately before the mineralization procedure, the freshly prepared solution containing ncHAp was treated with ultrasound for 30 s to prevent agglomeration. A titanium immersion probe and a Qsonica ultrasonic homogenizer were used (LLC, CT, USA), with 55W of transmitter power and an amplitude of 50%.

#### 2.6.3. Amino Acid Booster (AA)

AA includes a complex of low-concentration (up to 12%) saturated and unsaturated polyfunctional organic acids (maleic acid, polyacrylic acid, citric acid, distilled water) and polar amino acids (arginine: 0.2–1.6%, lysine: 0.05–0.4%, histidine: 0.01–0.2%). AA serves to activate the processes of deposition of HAp crystals of a given shape and their hierarchical organization on the surface of a pretreated biotemplate (tooth enamel).

### 2.7. Experimental Set-Up and Parameters

#### 2.7.1. Optical Microscopy

Optical images of the surface areas of samples N1–N5 were obtained using an Olympus CX40 optical microscope (Olympus, Tokyo, Japan). An Olympus 40 ×/0.65 plan achromat lens and a Levenhuk 5 MPix digital camera (Tampa, FL, USA) were used for imaging. The images were obtained from the central area of each sample in bright-field mode with flat-field correction. The size of the studied area on the surface of the samples was 300 × 400 μm at × 400 magnification. The images were analyzed using the ToupTek ToupView software package (Hangzhou ToupTek Photonics Co., Hangzhou, China).

#### 2.7.2. Atomic Force Microscopy

The surface morphology was studied using a Femtoscan-001 NT MDT scanning probe microscope. The samples were imaged at room temperature in air, in intermittent contact (semicontact), with a rectangular silicon cantilever, nominal elasticity constant k = 40 N/m, resonant frequency 40–440 kHz, and peak radius < 10 nm. After acquisition, the images were processed using Nova v1.1.0.1837 (NT-MDT, Moscow, Russia) software. The obtained AFM images were processed in order to remove the surface tilt and to align individual image lines. 

#### 2.7.3. Electron Microscopy

The surface morphology and cross sections of samples before and after mineralization were investigated with a combined Helios G4 UX focused ion beam scanning electron microscope (Thermo Fisher Scientific, Waltham, MA, USA) and field emission scanning electron microscope (FE-SEM, Quanta FEG 250, Hillsboro, OR, USA). SE (secondary electron) images were produced at 10 kV and 0.1 nA. 

#### 2.7.4. X-ray Diffraction

A study of the local crystal structure of the biomimetic mineralized layer and natural dental enamel was performed by X-ray diffraction using a DRON-8 thin-film X-ray diffractometer under the following conditions: Cu Kα1 radiation with a wavelength of λ = 1.54032 Å and an analyzed area of 100 × 100 µm^2^.

#### 2.7.5. Raman Microspectroscopy

Raman scattering was used to evaluate a chemical property of the biomimetically mineralized dental enamel tissue. A RamMix 532 Raman microscope (Enspectr, Moscow, Russia) was used with the following specifications: spectral resolution ~1 cm^−1^, laser excitation wavelengths 532 nm and 785 nm, radiation power ~30 mW, and signal collection with a 60× objective. 

Raman mapping was conducted using a motorized two-axis stage providing minimum step-by-step shifts of 300 nm. The area of the analyzed microregion was 1 × 1 μm.

#### 2.7.6. Data Collection and Spectral Processing 

Spectral processing (correction, averaging, integration, finding maxima) were performed using the Origin 8 software package (OriginLab Corporation, Northampton, MA, USA). 

Statistics were described using the SigmaPlot 13.0 software package (Systat Software Inc, San Jose CA, USA). Multivariate statistical analysis and chemometric analyses were performed using the CytoSpec (v. 2.00.07) software package.

#### 2.7.7. Measuring Nanohardness

The mechanical properties of the mineralized hard tissue were investigated by surface nanoindentation using a nanoindentation tester (CSM Instruments). To determine the hardness value, the Oliver and Farr method was used, which takes into account the nonlinearity of the onset of the unloading curve and offers a procedure for determining the contact depth based on the use of the indenter shape function and the indenter shape function conversion factor.

The maximum load on the indenter (Berkovich diamond pyramid) was 10.0 mH. For all the measurements, a linear mode of loading and unloading the indenter was used. Loading time was 45 s, holding time at the maximum load was 1 s, and unloading time was 30 s. At least 10 measurements were performed in each area and averaged. The results were processed using the indentation software package for CSM Instruments.

## 3. Results and Discussion

### 3.1. Microscopic Examinations

**Figure 2:**
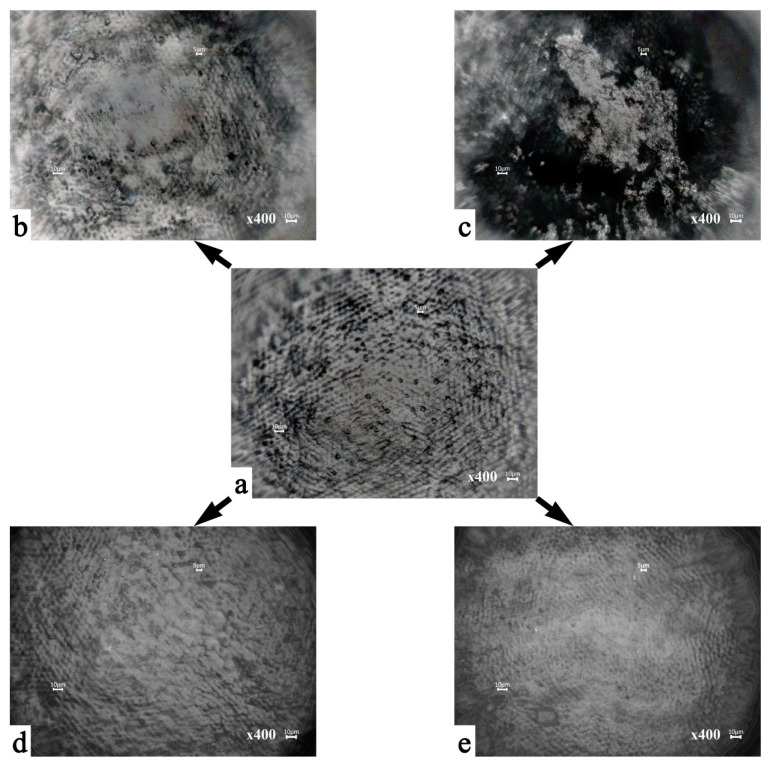
Optical images of the surface areas of the samples: (**a**) healthy tooth enamel without treatment (sample N1); (**b**) without pretreatment and with subsequent mineralization (sample N2); (**c**) after surface pretreatment with H_3_PO_4_ and subsequent mineralization (sample N3); (**d**) after surface pretreatment with Ca(OH)_2_ and subsequent mineralization (sample N4); (**e**) after surface pretreatment with Ca(OH)_2_ and amino acid booster, with subsequent mineralization (sample N5).

Figure 2 shows the optical images of the characteristic surface areas of the examined samples. A preliminary study of the samples of intact enamel mineralized layers using an Olympus optical microscope with 10×, 20×, and 40× plan achromat lenses demonstrated the absence of cracks and splits on the sample surfaces. 

In Figure 2a, a surface morphology typical of the lateral surface area of healthy enamel can be observed (sample N1), described in the literature as an “aprismatic surface layer” [5,24,39]. The main local elements of this morphology are enamel rods extending orthogonally to the enamel surface [5,24,39], between which there are protein-rich areas (a protein-rich sheath) or the so-called inter-rods space. The diameter of the observed enamel rods, based on the optical microscopy data presented, was ~5 μm, whereas the size of a typical interstitial space is ~2 μm (Figure 2). It should be noted that for the surface of a typical healthy enamel specimen (N1 samples), there is a specific distribution of the heights of the exiting enamel rods, which is evident from the change in contrast in the optical image (Figure 2a). It can clearly be seen that the surface of healthy enamel in the considered area has periodic recurrent height differences (up to 100 μm), which is a consequence of growth processes, Retzius lines, and natural cycles of demineralization and mineralization.

It should be noted that after pretreatment of the biotemplates using different approaches (Figure 1) and the subsequent mineralization, the surfaces of samples N2–N5 at the macro level show morphology areas typical of healthy enamel, as in N1 (Figure 2a–e). However, at the same time, images of the mineralized tissue show a different surface microrelief (morphological features at the microscopic level) depending on the pretreatment conditions.

Thus, mineralization of the enamel samples that were not pretreated (sample N2, Figure 2b), leads to deposition in some areas (from 10 to 100 μm^2^) on the surface of the biotemplate layer containing ncHAp. At the same time, there is no observed pattern in the formation of the microrelief in the mineralized area. We assume that the formation of similar areas of mineralization occurs by means of a spontaneous mechanism where crystallization points act as apatite crystals of natural enamel in places with the maximum height of relief (exit points of enamel rods).

Regarding the morphology of the mineralized layer in the samples that were pretreated with different agents (samples N3–N5, Figure 1), the analysis shows the formation on their surface of layers based on ncHAp with different organizations (devices). Thus, Figure 2c illustrates the typical surface morphology of the mineralized layer formed on a biotemplate that was pretreated with orthophosphoric acid (sample N3). It can clearly be seen that the formation of a surface relief with sharp height differences in the area of the enamel rods is characteristic of samples of this type. The formation of individual ncHAp-based agglomerates with typical sizes from 1 μm to 30 μm can be observed on the surface of these samples (Figure 2c). Regarding the morphology of the mineralized enamel surface after its pretreatment in a Ca(OH)_2_ solution (sample N4, Figure 2d), this type of sample is characterized by the formation of a more homogeneous relief. Uniform distribution of the deposited mineralized layer on the basis of ncHAp over the entire considered area can be observed.

In the case of the two-step pretreatment of a biotemplate (sample N5), first in an alkaline medium and then in an amino acid booster, the subsequent mineralization leads to a homogeneous distribution over the surface formed on the ncHAp mineralized layer without local inhomogeneities (Figure 2e). The surface does not exhibit sharp differences in height and corresponds best to the morphological organization of natural enamel (Figure 2a).

**Figure 3:**
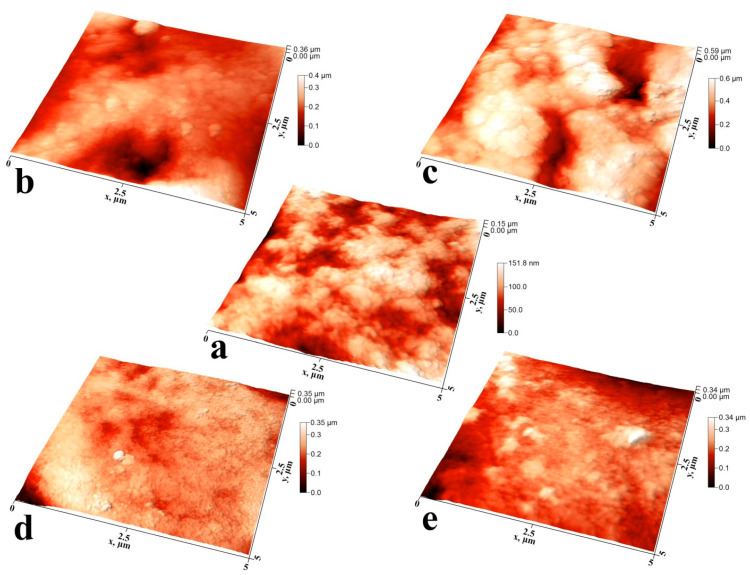
Three-dimensional AFM images of typical surface areas of mineralized tooth tissue samples: (**a**) healthy tooth enamel without treatment (sample N1); (**b**) without pretreatment and with subsequent mineralization (sample N2); (**c**) after surface pretreatment with H_3_PO_4_ and subsequent mineralization (sample N3); (**d**) after surface pretreatment with Ca(OH)_2_ and subsequent mineralization (sample N4); (**e**) after surface pretreatment with Ca(OH)_2_ and an amino acid booster, with subsequent mineralization (sample N5).

Studies of the individual characteristic microplots of mineralized tissue samples using atomic force microscopy allowed us to study the features of the formed surface microrelief during ncHAp deposition (Figure 3a–e).

Figure 3a shows an AFM image of a typical surface of an intact enamel sample (sample N1) with a 5 × 5 μm area. The surface morphology has a characteristic microrelief and nanorelief due to the complex hierarchical structure of enamel. The microrelief is determined by the existence of areas of enamel rods and the space between them. Each of the enamel rods consists of a grouping of oriented hydroxyapatite nanoprisms bonded together by a protein matrix [5,7,24,39]. The characteristic thickness of enamel prismatic nanocrystals is ~20 nm. However, the surface of natural enamel (Figure 3a) has been subjected to natural acts of mineralization and demineralization over time, resulting in larger agglomerates of ~30 nm [40].

It should be noted that the surface of the healthy enamel sample (sample N1) has a local roughness associated with the exit rods on the enamel surface, as well as the array of apatite nanocrystals forming them. Large height differences across the surface are associated with non-uniformity of natural mineralization of the natural enamel surface and the hierarchical organization of apatite nanocrystals within the enamel rod and in the inter-rod regions [40]. 

**Figure 4:**
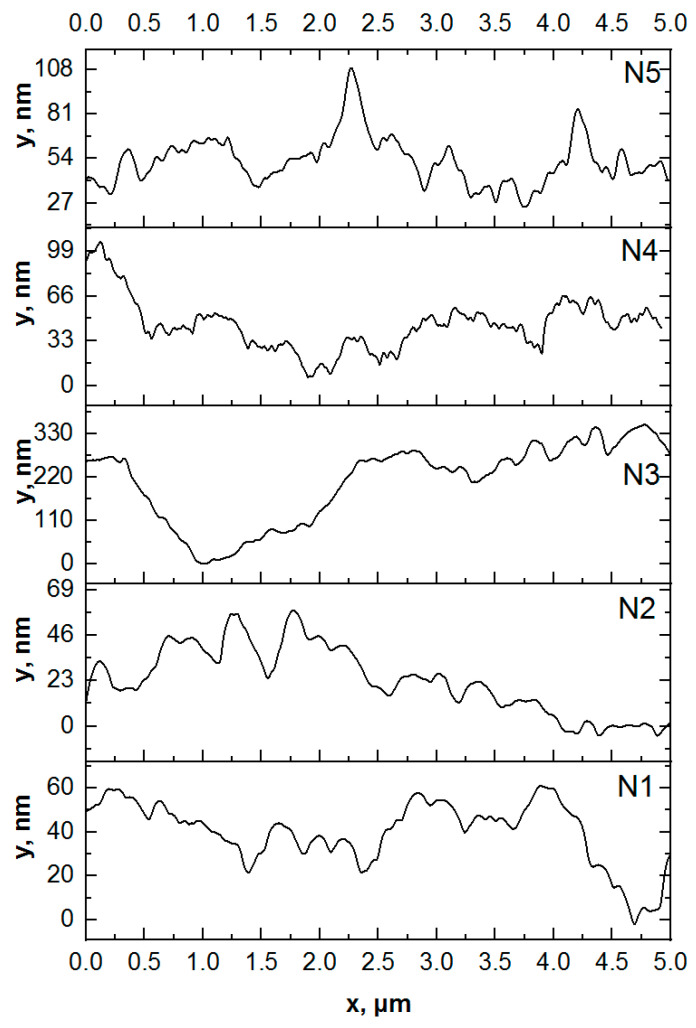
Line-cut profiles of AFM topography images: sample N1: healthy tooth enamel without treatment; sample N2: without pretreatment and with subsequent mineralization; sample N3: after surface pretreatment with H_3_PO_4_ and subsequent mineralization; sample N4: after surface pretreatment with Ca(OH)_2_ and subsequent mineralization; sample N5: after surface pretreatment with Ca(OH)_2_ and an amino acid booster, with subsequent mineralization.

It should be noted that the mineralization of enamel without pretreatment (sample N2) changes the morphology and substructure of the surface layer. For this type of specimen, we observe local areas of mineralization, detected previously in optical images (see Figure 2), consisting of large agglomerates of apatite nanocrystals ~100 nm in diameter. This exceeds both the size of the ncHAp used for mineralization [30] and the width of the enamel apatite nanoprism ends oriented normally to the surface in the enamel rods area. The appearance of such agglomerates on the surface can be caused by the use of a weakly alkaline solution in which the crystallization of hydroxyapatite occurs during the mineralization of enamel. A comparison of profiles of the AFM morphology for samples N1 and N2 (see Figure 4) clearly demonstrates the presence of a greater number of large agglomerates on the surface of sample N2.

Pretreatment of the biotemplate in a phosphoric acid solution (sample N3) resulted in the formation of a specific surface morphology, with etching of the enamel rods at a higher rate than in the interstitial area [41]. AFM topography analysis of samples of this type revealed the presence of areas of inhomogeneous mineralization, with weak adhesion to the enamel and a height difference of more than ~350 nm (Figure 3c and Figure 4). It can clearly be seen that on the surface of sample N3 (see Figure 3c), there are large (from 100 to 300 nm), chaotically located agglomerates on the mineralized layer. The appearance of such heterogeneities (calcium-phosphate agglomerates) on the enamel surface indicates the presence of broken bonds and the formation of acidic phosphates as a result of biotemplate etching [41] and subsequent mineralization in a slightly alkaline solution.

Biotemplate pretreatment using an alkaline solution resulted in a more homogeneous micromorphology of the mineralized layer, with the presence of texture on the entire surface. Thus, for sample N4, AFM confirms the formation of a more homogeneous mineralized layer on the surface simultaneously with the presence of agglomerates with a fine structure (see Figure 3d and Figure 4). Analysis of the line-cut profile of the AFM topography for samples of this type (N4) reveals the presence of nanoparticles with an average size of 35 nm, homogeneously distributed on the surface of the mineralized layer. This value is comparable with the typical size of hydroxyapatite ncHAp nanocrystals used to create the mineralized layer. The formation of such a morphology is due to the fact that ncHAp nanocrystals are oriented orthogonally to the surface of the biotemplate and are spliced along the borders of grains.

The surface morphology of sample N5, which is characterized by features similar to those of sample N4, is worthy of special attention, The formation of larger particles (~70 nm in size) as a result of the splicing of ncHAp nanocrystals into agglomerates is observed on the surface. This behavior may be a consequence of the additional enamel treatment with an amino acid booster solution, which, as shown in a number of studies [13,42], promotes the directed aggregation of nanocrystalline hydroxyapatite.

**Figure 5:**
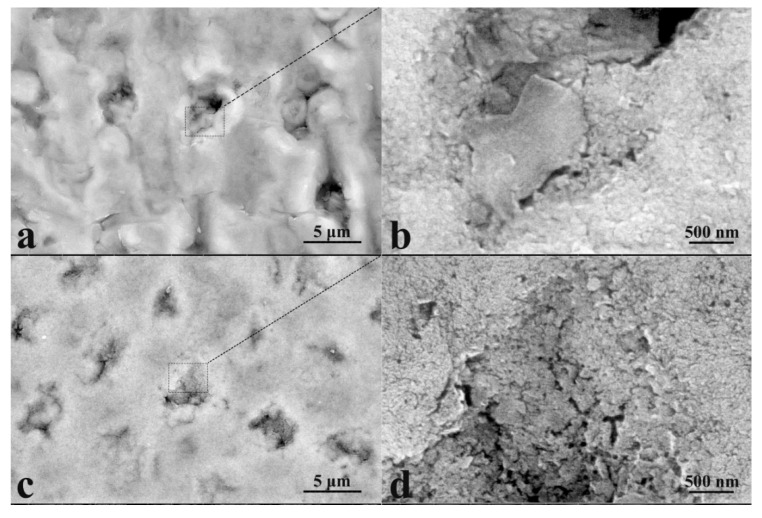

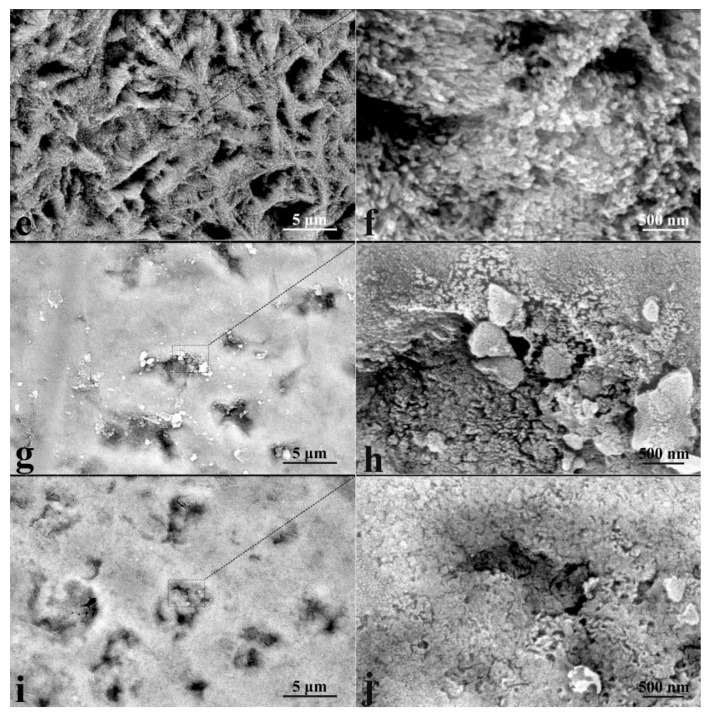
FESEM images of typical surface areas of mineralized tooth tissue samples with two magnifications (×13,000 on the left and ×50,000 on the right): (**a**,**b**) healthy tooth enamel without treatment (sample N1); (**c**,**d**) without pretreatment and with subsequent mineralization (sample N2); (**e**,**f**) after surface pretreatment with H_3_PO_4_ and subsequent mineralization (sample N3); (**g**,**h**) after surface pretreatment with Ca(OH)_2_ and subsequent mineralization (sample N4); (**i**,**j**) after surface pretreatment with Ca(OH)_2_ and amino acid booster, with subsequent mineralization (sample N5).

The formation of mineralized layers is also confirmed by the results of electron microscopy. Additional information on the morphology of the surface mineralized layers was obtained with the use of a focused ion beam scanning electron microscope. Typical images of the surface areas of the mineralized tissue for the investigated samples are presented with different magnifications in Figure 5. Based on the obtained results, the SEM data are in good agreement with the results of AFM imaging, and in addition they allow us to visualize the features of the morphology in the mineralized tissue, accounted for by different techniques of biotemplate pretreatment. 

For example, Figure 5a,b demonstrate the surface of healthy enamel (sample N1) with a characteristic aprismatic surface layer, while at the macro level one can see enamel rods and inter-rods space. Note that after the mineralization procedure, it is possible to see morphological areas, just as in the AFM images, which are characteristic of healthy enamel (see Figure 3). It is clearly observed that on the surface of the samples after their mineralization, there exists a layer formed by nanocrystals, and their size is comparable with that of hydroxyapatite (ncHAp) nanocrystals (50−70 nm). One can distinctly see that the surface morphology of the mineralized layer for sample N5 (after surface pretreatment with Ca(OH)_2_ and amino acid booster) is very close to the morphology of native enamel. At the same time, the treatment of enamel with an acid (sample N2) not only results in local surface mineralization (for AFM results, see Figure 3c) but also prevents the duplication of the features of the native tissue in the mineralized layer. 

**Figure 6:**
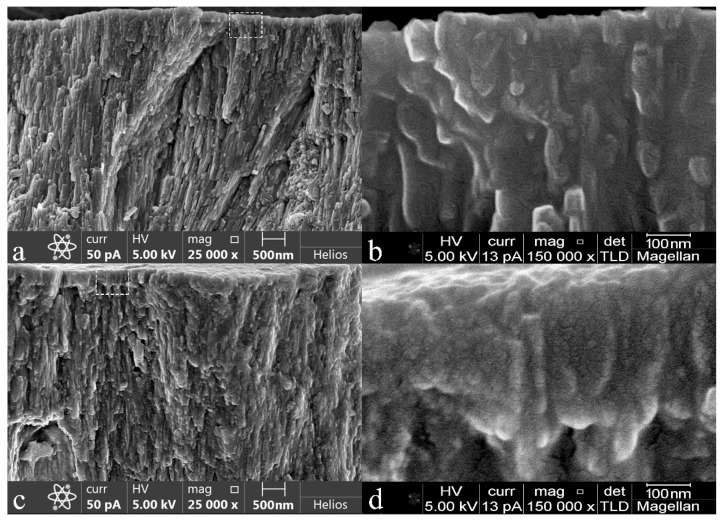
Typical cross-section SEM images of mineralized tooth tissue: (**a**,**b**) healthy tooth enamel without treatment (sample N1); (**c**,**d**) after surface pretreatment with Ca(OH)_2_ and an amino acid booster, with subsequent mineralization (sample N5).

Cross-section studies of the native enamel sample (sample N1) and enamel with a mineralized layer (sample N5), including those with a greater magnification of ×150,000, both confirm and visualize the sizes of the formed mineralized layer (see Figure 6). It can clearly be seen that for the sample of native enamel (sample N1), the cross-sectional SEM image quite obviously demonstrates the local hierarchy of the enamel pathways, with a predetermined direction of apatite nanocrystals orthogonally to the surface within the enamel. Figure 6b, with a high-resolution microphotograph of a region of the cross section of size ~ 500 × 800 nm, shows that apatite nanocrystals of the intact enamel have a smaller length and less ordering compared to the internal layers, which is due to the processes of natural crystallization. 

In the case of sample N5 (Figure 6c,d), the formation of a mineralized layer comprising particles and agglomerates of ~50 nm in size is observed on the surface, which corresponds to the sizes of ncHAp nanocrystals [31] used for the layer formation. The thickness of this layer varies from 150–200 nm, while the direction of some ncHAp nanocrystals coincides with that of apatite crystals in the enamel. This result correlates quite well with the AFM studies of the mineralized layers.

**Figure 7:**
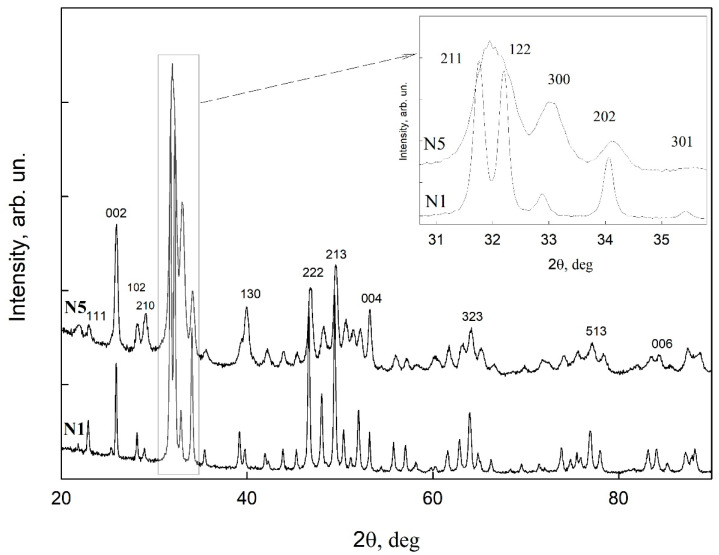
Results of XRD analysis of healthy tooth enamel without treatment (sample N1) and the sample with mineralized layer obtained after surface pretreatment with Ca(OH)_2_ and an amino acid booster, with subsequent mineralization (sample N5).

In order to confirm the phase composition of the mineralized layers on the surface of the biotemplates, we used an X-ray diffraction technique. The results of X-ray microdiffraction obtained with the sliding beam geometry for sample N1 (healthy tooth enamel without treatment) and sample N5 (surface pretreatment with Ca(OH)_2_ and an amino acid booster, with subsequent mineralization) are presented in Figure 7. The latter contains a homogeneous mineralized layer, according to the microscopy data. According to the obtained experimental data (Figure 7), it can clearly be seen that the diffractograms of both samples show the same set of reflections, with Miller indexes corresponding to ncHAp (ICDD #01-089-6438) [43], used in our work for the formation of a mineralized layer as a basis for the native artificially mineralized tissue. Neither of the additional phases can be observed in the diffractograms, meaning that the mineralized layer grown on the biotemplate surface is really hydroxyapatite and not any other phosphate which is similar in structure to hydroxyapatite. Moreover, as seen from the inset to Figure 7, hydroxyapatite crystals of the mineralized layer (sample N5) have a predominant orientation relative to the ordered direction of HAp crystals in the apatite of intact enamel (i.e., perpendicular to the enamel–dentin boundary). This can be observed by the change in intensity of the brightest lines ((211), (300), and (112)) in the diffractograms of the samples. 

It should be noted that the broadening of reflections in the diffractogram of sample N5 compared to sample N1 (intact tooth enamel) is due to the contribution of diffraction from the layer and a substrate, as well as dimensional and concentration factors. The dimensional factor is associated with the fact that in order to produce a mineralized layer, we used nanocrystalline HAp with a typical particle size of ~35nm, which can form larger agglomerates, according to the AFM data. However, the characteristic sizes of enamel apatite are in the sub-micrometer range, thus resulting in narrower reflections in the diffractogram of native enamel. Regarding the concentration factor, the presence of a slight gradient in the composition of the deposited bioinspired apatite leads to a broadening of the diffraction lines, while the change in lattice parameters results in the observed shift of the diffraction lines (see inset to Figure 7). 

### 3.2. Raman Microspectroscopy

The structure of dental enamel has specific crystallochemical properties, in particular the characteristic atomic Ca/P ratio, the percentage of carbonate anions CO_3_^2−^ in the crystal lattice, and the characteristic B-type substitution, where the carbonate anion CO_3_^2−^ is included in the position of the PO_4_ group [34]. Therefore, we studied the differences in the chemical composition between healthy natural and artificially mineralized tissue, taking into account the hierarchical features of enamel structure, using Raman microspectroscopy, which is sensitive to distortions in the crystal structure of apatite [44], orientation [45], and nanocrystal size [46].

**Figure 8:**
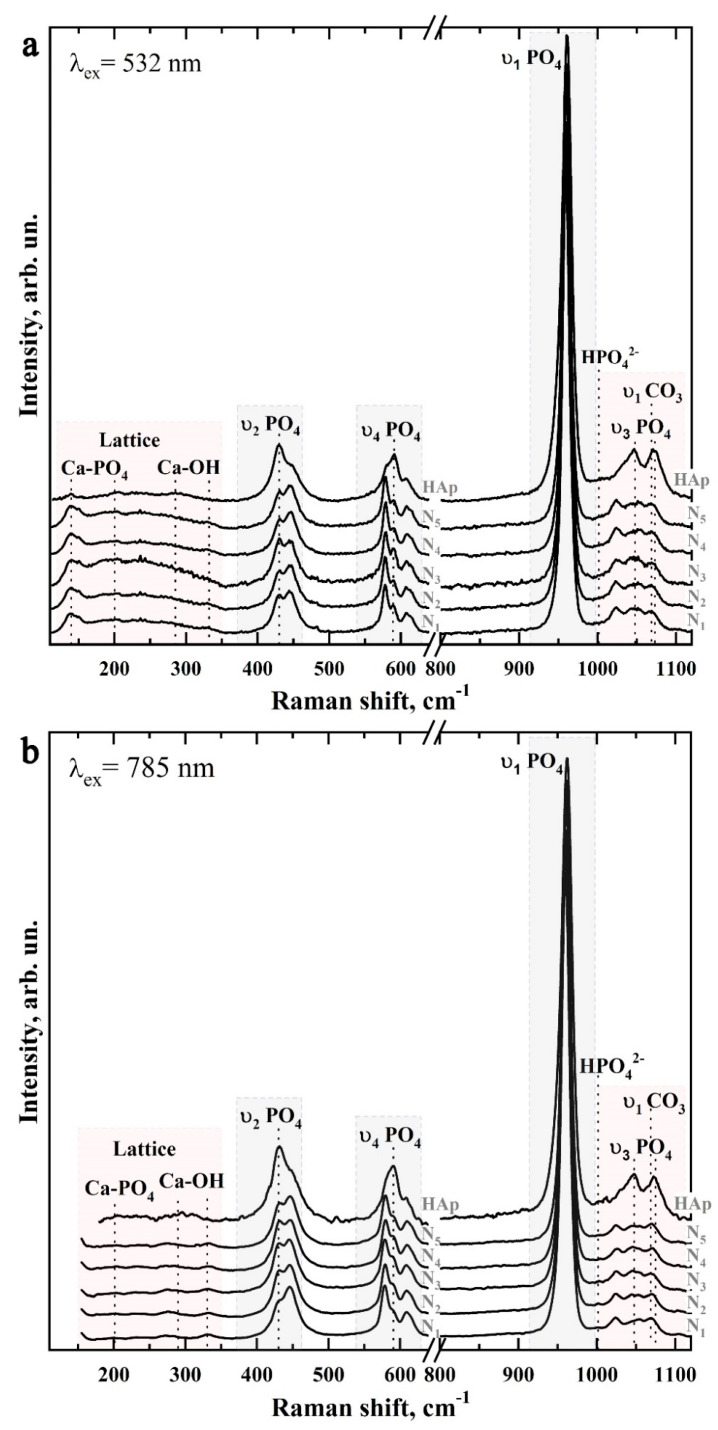
Typical Raman spectra of the studied samples: (**a**) excitation using 532 nm laser radiation; (**b**) excitation using 785 nm laser radiation.

Figure 8 shows typical Raman spectra of mineralized tissues (samples N1–N5), as well as the spectrum of the nanosized carbonate-substituted calcium hydroxyapatite used. The spectra are presented after baseline correction without normalization. Raman spectra were obtained by using two sources of laser radiation: 532 nm (Figure 8a) and 785 nm (Figure 8b) and are presented in the 100–1150 cm^−1^ range, in which the main vibrations associated with the mineral component of the samples are located. 

It should be noted that most often, for the registration of a Raman signal of apatite-containing tissues, radiation with a wavelength of 532 nm is used. However, this leads to an intensive fluorescence background, which affects the quality of the spectra. Simultaneous use of the second excitation wavelength of 785 nm allowed us to partially eliminate the fluorescence background and to observe a useful signal in the lattice region of 150–350 cm^−1^ and small intense vibrations in the region of 1000–1020 cm^−1^. However, due to the peculiarities of the experimental scheme, when Raman scattering was excited using the 785 nm laser, the signal registration started from 150 cm^−1^.

The preliminary analysis of the experimental data showed that the Raman spectra within the chosen group of samples contained a similar set of modes, which differed in intensity at different scanning points. Therefore, the Raman spectra presented below are averaged in groups.

**Figure 9:**
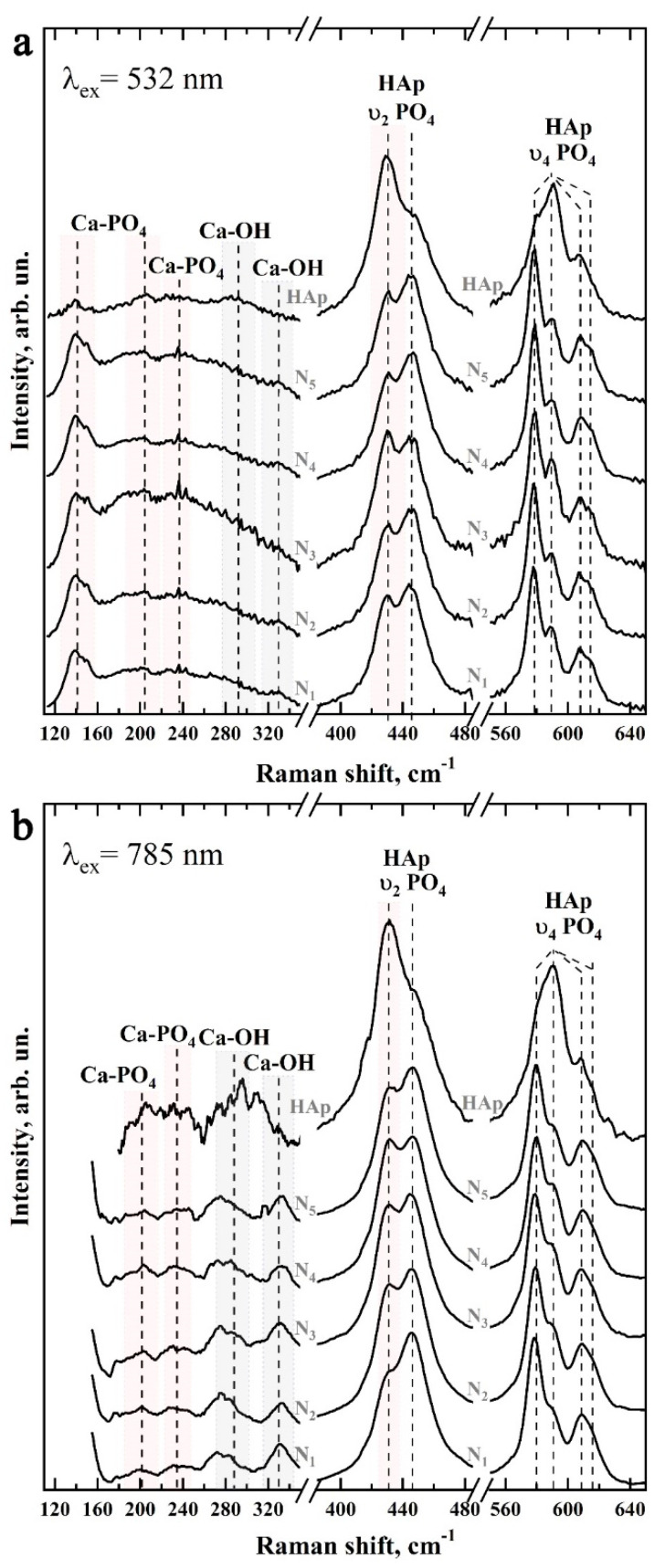
Raman spectra of samples N1–N5 and ncHAp in the regions 120–330 cm^−1^ (left), 400–480 cm^−1^ (center), and 560–640 cm^−1^ (right): (**a**) excitation using 532 nm laser light; (**b**) excitation using 785 nm laser light.

**Figure 10:**
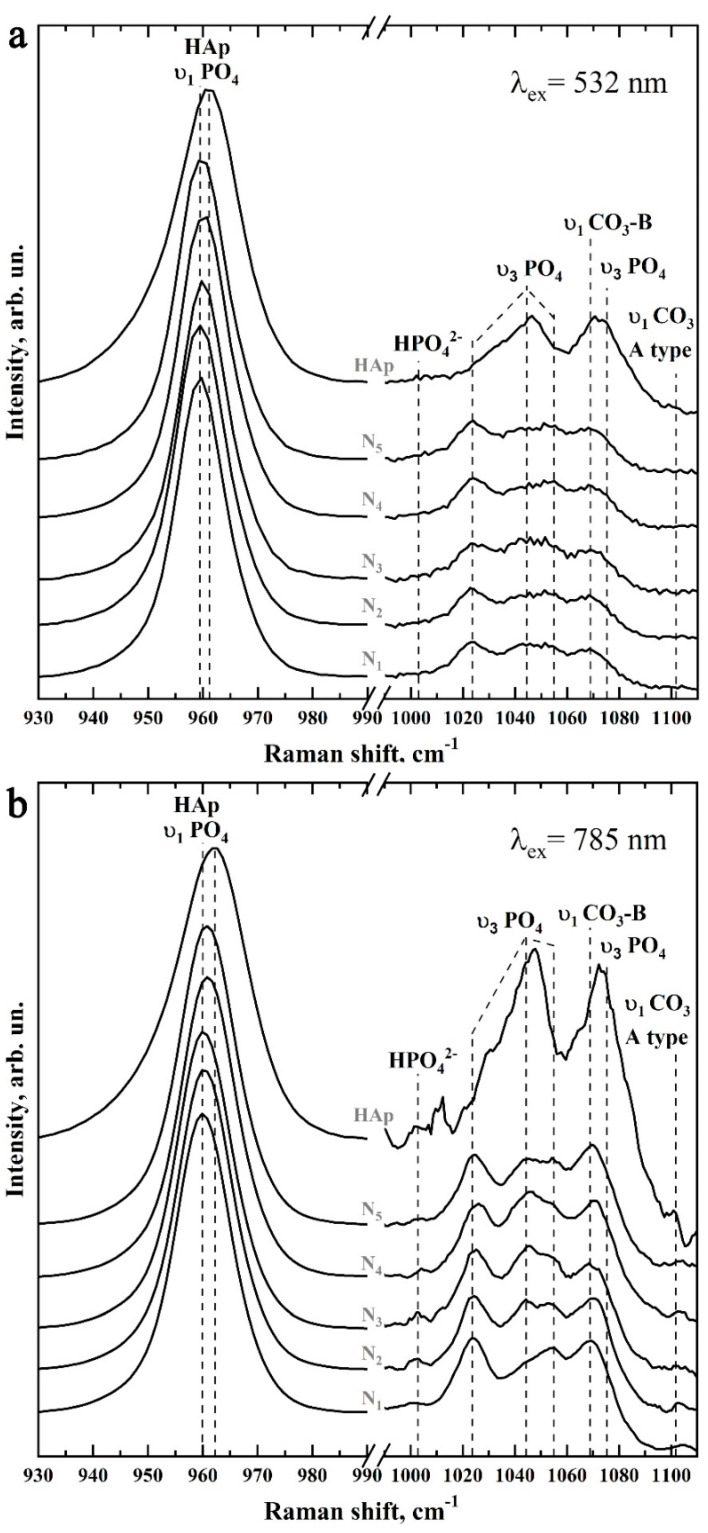
Raman spectra of samples N1–N5 and ncHAp in the regions 930–990 cm^−1^ (left) and 990–1110 cm^−1^ (right): (**a**) excitation using 532 nm laser radiation; (**b**) excitation using 785 nm laser radiation.

Figure 9 and Figure 10 show in more detail the five spectral regions in which the main spectral features characteristic of the studied samples are located.

It is well known that the main components of mineralized natural dental tissue (enamel) are phosphates, carbonates, and components of the protein amelogenin [5]. The analysis of the results shows that the basic and most intensive modes in the spectra of samples of artificially mineralized and natural tissues, and also of synthesized bioinspired apatite, can be attributed to characteristic vibrations of carbonate-substituted calcium hydroxyapatite (see Table 1), which is a basis of the mineral component of the investigated samples [45,47,48,49,50,51].

Thus, phosphate ions are associated with four groups of active bands in the Raman spectra of both mineralized tissue and nanocrystalline hydroxyapatite ncHAp. The most intensive maximum here is the υ_1_ symmetric valence vibration of PO_4_^3−^, localized in the region of 930–990 cm^−1^ [45,47,48,50,51,54]. It is necessary to pay attention to the different positions of the main maximum in the spectra of the studied samples. Thus, in the spectrum of the biomimetic nanosized carbonate-substituted calcium hydroxyapatite ncHAp, the υ_1_ PO_4_^3−^ mode has a maximum at about 961.5 cm^−1^, while in the spectrum of natural enamel this peak is shifted to 959.5 cm^−1^, which coincides with the already known literature data [50,60,61].

The next group of bands attributed to phosphate ions are the less-intense υ_3_ stretching oscillations, consisting of three overlapping maxima around 1025 cm^−1^, 1041 cm^−1^, and 1045 cm^−1^, and the υ_2_ and υ_4_ PO_4_^3−^ bending modes. The υ_2_ PO_4_^3−^ bending mode appears as two well-resolved peaks at ~430 cm^−1^ and 445 cm^−1^, while the υ_4_ PO_4_^3−^ mode appears as a group of four overlapping maxima at ~579 cm^−1^, 590 cm^−1^, 609 cm^−1^, and 616 cm^−1^ [45,47,48,50,51,54]. It should be noted that the intensity and position in the Raman spectrum of the modes associated with PO_4_^3−^ phosphate ions also depend on the sample type (see Figure 9 and Figure 10).

Regarding the carbonate groups present in the structure of apatite, in the Raman spectra they are associated with the active vibrational band υ1, which occurs due to the inclusion of carbonate anions CO_3_^3−^ in the crystal lattice of calcium hydroxyapatite. In this case, when the carbonate anion of the PO_4_^3−^ group is substituted (B-type substitution, typical for biogenic materials [47,49,51,57,58,62]), a maximum of about 1068–1070 cm^−1^ is observed in the spectrum (Figure 10). At the same time, the inclusion of carbonate anions CO_3_^3−^ in the lattice instead of the OH-group of apatite (A-type substitution, characteristic of natural enamel [47,49,50]) leads to the appearance of a peculiarity in the range of 1001–1104 cm^−1^ (Figure 10).

Another low-intensity oscillation appearing at 1003 cm^−1^ in the spectra of mineralized tissue and nanoHAp (Figure 10) can be ascribed to the acidic phosphate ions HPO_4_^3−^ contained in the samples. It should be noted that, as noted in G. Penel et al. [47], this fluctuation is characteristic of well-oxidized apatites (such as the enamel of the tooth) containing HPO_4_^3−^ ions. However, in synthetic carbonate-substituted apatites obtained under alkaline conditions, this band was not observed. The appearance of this fluctuation in the spectra of the mineralized hard tissues (samples N1–N5) used for the mineralization of the nanosized carbonate-substituted calcium hydroxyapatite ncHAp (Figure 10) could be a consequence of the fact that the last sample was synthesized under weak alkaline conditions [34].

Another group of modes of inclusive interest in Raman spectra, associated with lattice vibrations as well as isolated ions [44,52,53] in natural and mineralized hard tissue, is located in the 110–330 cm^−1^ region (see Figure 9). Registration and analysis of the spectral features associated with lattice oscillations can offer important additional information about the processes which take place during solid-tissue mineralization and are accompanied by substitutions in the crystal structure of apatite [63], as well as changes in the coordination environment of calcium atoms [34,52,63].

As noted above, the intense background fluorescence when excited by Raman scattering using 532 nm radiation does not allow for the resolution of lattice oscillations in this region (see Figure 9a and Figure 10a). However, these modes are easily distinguishable in the spectra when excited by a 785 nm laser (see Figure 9b and Figure 10b). The analysis of the obtained data shows that the maxima in the Raman spectra approximately located around 140 cm^−1^, 200 cm^−1^, 233 cm^−1^, and 284 cm^−1^ can be ascribed to the Ca-PO_4_ bound vibrations of the apatite lattice. Similarly, vibrations around ~188 cm^−1^, 273 cm^−1^, 312 cm^−1^, and 330 cm^−1^ are associated with Ca_II_-(OH) bonding [53,63]. In our previous work [34], it was shown that it is more typical for the surface of ncHAp nanosized calcium carbonate-substituted hydroxyapatite crystals to have a calcium atom in the Ca_II_ position associated with the hydroxyl OH group. In addition, since in the crystal lattice of hydroxyapatite the Ca_II_-(OH) bonds represent ~60% of the total, the activity of the experimentally observed modes in the Raman spectra is natural.

The redistribution of intensities and changes in the position of the bands observed in the Raman spectra of samples during the formation of mineralizing layers using nanosized carbonate-substituted calcium hydroxyapatite and the appropriate type of pretreated biotemplate are reflected in the shape of the overall profile of Raman scattering in the vibration region υ_2_–υ_4_ PO_4_^3−^ (see Figure 9). It can be noted that the most similar are the band profiles of the natural enamel (sample N1) and the sample with the mineralized layer (sample N5) obtained using an alkaline solution of Ca(OH)_2_ and an amino acid booster (AA). 

To establish the influence of the biotemplate pretreatment method on the chemical differentiation and spatial distribution of the mineralizing layer, we performed Raman mapping of characteristic 5 µ × 5 µ surface areas of samples N1, N2, N4, and N5, taking into account the morphology of the enamel surface. For this purpose, an automated motorized two-axis stage was used, providing a minimum incremental displacement of 250 μm. The selected sample sections were scanned with a spatial resolution of 0.5 µm, and signal acquisition was performed with a 100× objective lens. The spectrometer was carefully calibrated beforehand using the silicon Raman mode. To increase the signal-to-noise ratio, the spectrum at each map point was averaged based on 40 scans.

Chemical imaging (functional group mapping) of the surface areas was then performed using the CytoSpec software package. As a result, chemical images were created, which are distributions in the color scale of the values of the ratio of intensities in the selected frequency range. For chemical imaging, we chose the ratio of the integral intensities of the low-frequency and high-frequency components of the υ_2_ PO_4_^3−^ doublet:

(1) $$R=\frac{\left\{405–435\right\}{\mathrm{cm}}^{-1}}{\left\{435–480\right\}{\mathrm{cm}}^{-1}}$$

**Figure 11:**
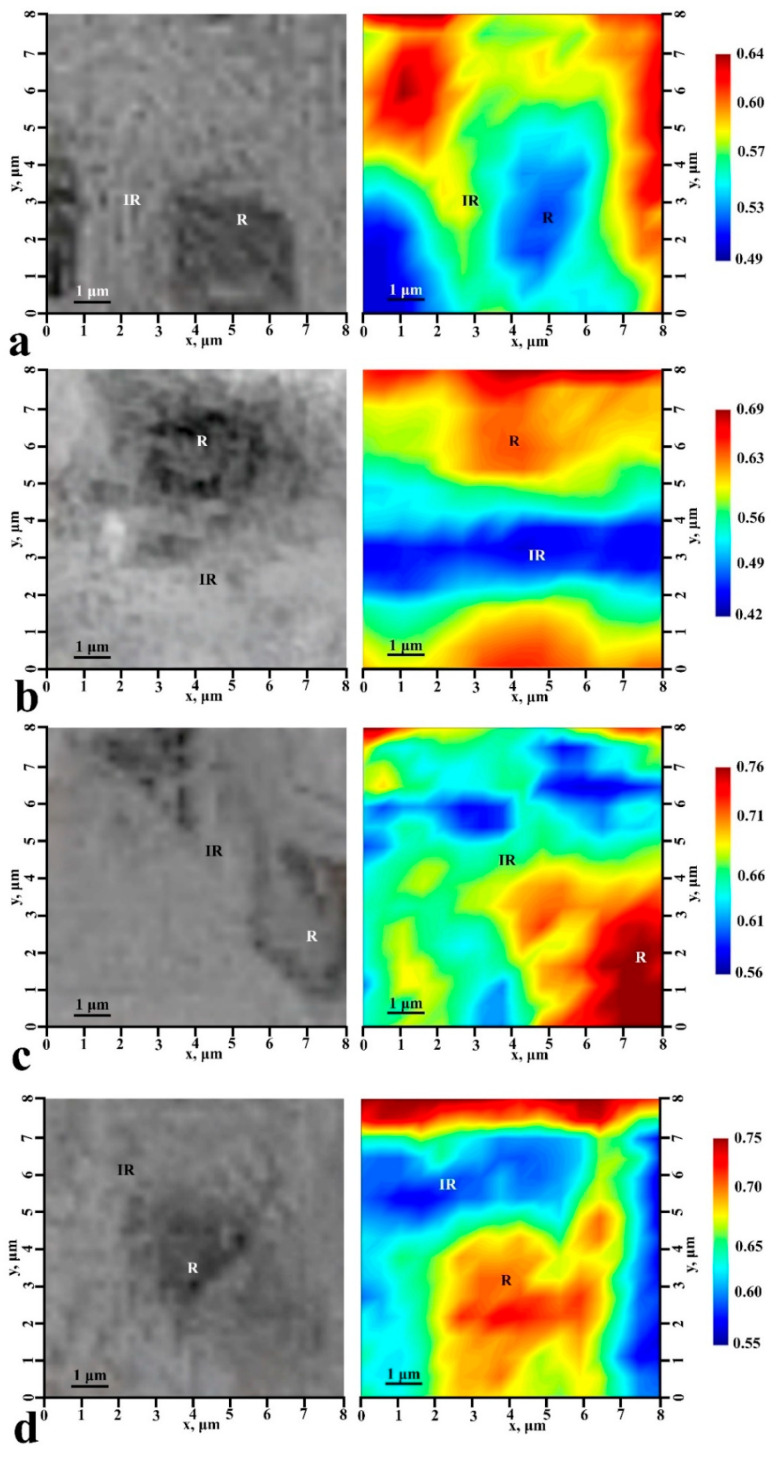
Optical (left) and chemical (right) images of the typical sample surface areas: (**a**) healthy tooth enamel without treatment (sample N1); (**b**) without pretreatment and with subsequent mineralization (sample N2); (**c**) after surface pretreatment with Ca(OH)_2_ and subsequent mineralization (sample N4); (**d**) after surface pretreatment with Ca(OH)_2_ and amino acid booster, with subsequent mineralization (sample N5).

The obtained typical chemical maps, as well as optical images of the microsurfaces of samples N1, N2, N4, and N5 for which mapping was performed, are shown in Figure 11. Note that we do not give the chemical image for a typical sample of N3 in Figure 11, because it was already noted earlier that the chosen method of biotemplate pretreatment did not allow us to create a uniform mineralizing layer and led to the formation of accumulations of particles of hydroxyapatite on the surface.

The analysis of the chemical imaging maps shows that the value of the R-ratio chosen for chemical imaging depends not only on the location on the surface of the mineralized tissue, and thus on the chemical composition at a particular point, but also on the type of biotemplate pretreatment. Thus, for healthy enamel (sample N1) this ratio varied from 0.49 to 0.64. At the same time, in the area of the center of the enamel rods, R takes the minimum possible value, and in the inter-rods area, where the deviation from the orientation of local crystallites is great [64], this ratio is a maximum.

A totally opposite picture is demonstrated by samples N2, N4, and N5. The R-ratio in the chemical images is maximal in the area of the center of the enamel rods and minimal in the interstitial area (Figure 11b–d). At the same time, in typical areas of samples N4 and N5, the R-ratio is higher, lying in the range 0.55–0.75. The reason for this, in our opinion, is not only the active growth of ncHAp crystals, for which the integral intensity of the low-frequency satellite υ_2_ PO_4_^3−^ doublet is significantly higher than that of the high-frequency satellite (see Figure 9), with R~1.32, but also their preferential orientation (texture) appearing in case of pretreated biotemplate surfaces. However, for all that, the chemical imaging maps allow us to visualize the features of the mineralized tissue surface morphology for all the samples in a similar way to optical images (Figure 11).

The values of the R-ratios in the area of the center of the enamel rods and the interstitial area of the samples are shown in Table 2.

### 3.3. Measuring Nanohardness

**Figure 12:**
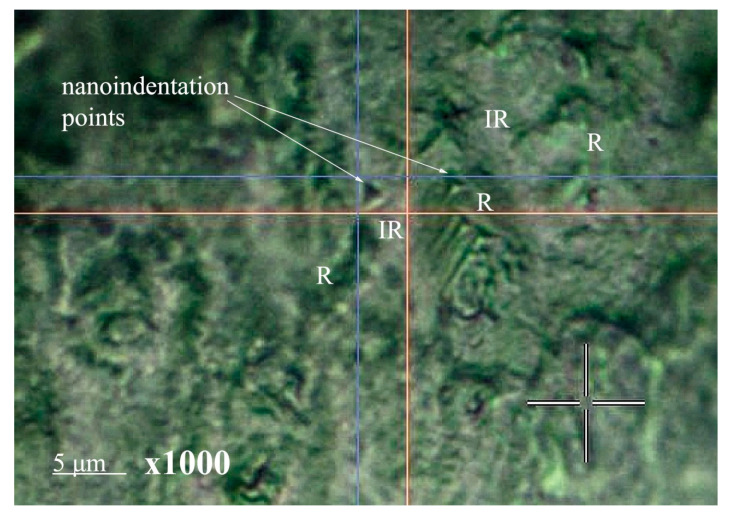
Typical image of the surface area of mineralized dental tissue, showing the enamel rods and inter-rods areas, as well as the nanoindenter prism imprint. R: the enamel rods areas; IR: the enamel inter-rods areas.

Since the hierarchical enamel substructure is formed by oriented enamel rods consisting of tightly packed hydroxyapatite nanoprisms bound together by protein and peptides, nanoindentation of natural and mineralized tissue surfaces was performed both in the area of enamel rods and in the adjacent inter-rods area (see Figure 12). Tests were performed at several points on the characteristic surface areas, and the direction of indentation was perpendicular to the surface of the mineralized tissue samples. Figure 12 shows an image of the enamel surface on which the surface morphology features (enamel rods and inter-rods areas) and an imprint of the nanoindenter prism are clearly visible. It should be noted that the choice of optimal nanohardness measurement parameters corresponded to the size of the indenter imprint (~1 μ), which in turn is smaller than the diameter of a typical enamel rod (~5 μ). This confirms our assumption that the nanoindentation impact of the prism occurred within a single rod or inter-rod region.

As a result of nanoindentation, the Vickers hardness was determined from the recorded load–displacement curve for each indentation test for samples N1, N2, N4, and N5 in the enamel rods and inter-rods areas. The hardness was determined based on at least 10 measurements at different points for each of the five samples included in the group. Subsequently, the results were averaged and presented in Table 2 as the mean value ± standard deviation. Then, the obtained results were averaged by sample type. Hardness measurements were not performed for sample N3 due to morphology features (presence of significant surface variations), inhomogeneities, and poor adhesion during formation of the mineralized layer (see microscopic results, Figure 2 and Figure 3).

**Figure 13:**
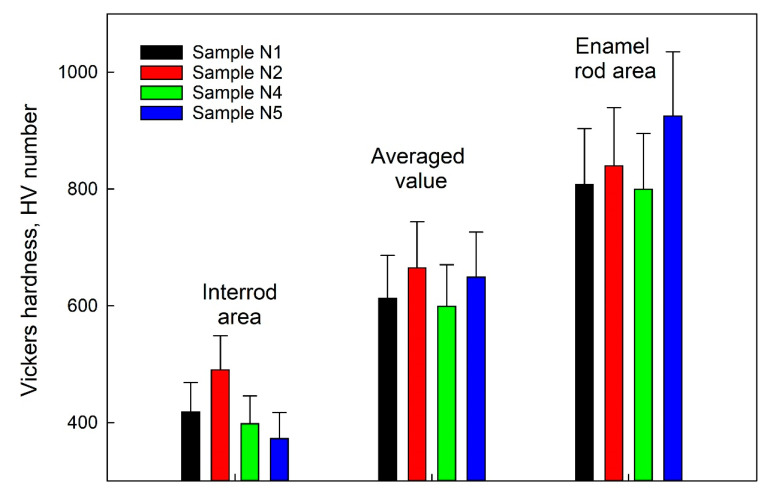
Vickers hardness in units of HV for the studied samples in the enamel rods area and inter-rods area, as well as the averaged value for a particular type of sample. The diagram shows the mean value of the Vickers hardness and the standard deviation.

Figure 13 shows a bar chart indicating the Vickers hardness in HV units for the studied samples in the enamel rods region and the inter-rods region, with the averaged value for a given sample type. The diagram also shows the standard deviation values. 

It follows from the obtained results that the averaged value of the hardness of the surface layer of a healthy enamel sample (N1) takes a value of HV of ~600, which corresponds to the data known from the literature [64]. Thus, the hardness value in the area of the enamel rods is almost double that of the inter-rods area, which is due to the orientation of the apatite nanocrystals of natural enamel.

The measured hardness values of N2 samples (without biotemplate pretreatment) exceeded those of healthy enamel (samples of type N1). This fact is associated with the aggregation and formation on the surface of N2 samples of local areas of mineralization (Figure 2b). Analysis of the AFM images of these areas (Figure 3b) shows that they are large agglomerates of HAp, significantly exceeding the size of apatite crystals of enamel. Moreover, according to the AFM data, the size of the mineralized layer is ~250 nm. Such an agglomeration of HAp on the surface of a biotemplate (enamel) results in the multiple conjugation of individual blocks and nanocrystals and their binding to the enamel. Therefore, the hardness value in the area of the enamel rods and in the interstitial area in sample N2 is higher than in similar areas of healthy enamel (sample N1).

Regarding mineralized tissue samples formed on pretreated substrates (samples N4 and N5), pretreatment of the enamel surface with Ca(OH)_2_ alkaline solution and polar amino acid solution resulted in decreased hardness of the mineralized layer in the interstitial region. For sample N4 (using only an alkaline solution), the hardness value in the area of the enamel rods was at the level of healthy enamel (sample N1). However, the simultaneous use of an alkaline solution and an amino acid booster for biotemplate pretreatment (sample N5, Figure 13) significantly (>15%) increased the Vickers hardness value in the enamel rod area compared to that of natural enamel (sample N1).

Since the strength characteristics (modulus of elasticity) of thin layers are determined primarily by chemical bonds, the observed change in the HV hardness of samples by means of different methods of pretreatment and mineralization of enamel can be explained both by a composition gradient in the formed mineralized layer in relation to natural enamel and by variations in the orientation of nanocrystals in both the rods and the inter-rods regions [64]. Pretreatment of the biotemplate in an alkaline solution (sample N4) led to a change in the morphology of the enamel surface layer. Subsequent mineralization of samples of this type led to the formation of a layer with homogeneous morphology on the surface (Figure 2d and Figure 3d). According to AFM and SEM data, on the surface of sample N4, a homogeneous fraction of hydroxyapatite is deposited, consisting of nanocrystals of ncHAp and their small agglomerates, which are comparable with the lateral dimensions of apatite nanocrystals in enamel (Figure 3d). As a result, no aggregation occurs on the surface of samples of this type, as in the case of sample N2, and the ncHAp crystal splits the mineralized layer into separate blocks along the planes of adhesion when indented, which is also characteristic of intact enamel [64,65]. The lower hardness value for the mineralized layer in sample N4 can be explained by the absence of a protein or peptide component at the boundaries of agglomerates and mineralized layer nanocrystals, which in intact enamel provides the necessary binding of the mineral component. It is for this reason that in our work, an amino acid booster was used during the pretreatment stage of sample N5. The chosen set of amino acids is characteristic of the natural enamel matrix, has already demonstrated its effectiveness in the formation of hybrid layers [33] and in directing the mineralization of hydroxyapatite [14,19,20], and can be used for ncHAp co-precipitation on biotemplate surfaces (tooth enamel) [31].

Amino acids not only allow the binding of HAp nanocrystals but also contribute to a certain morphological organization. The role of the alkaline environment in the formation of the amino acid–hydroxyapatite bond has been demonstrated in a number of relevant studies [42,66,67]. For this reason, biotemplate treatment with an amino acid booster and subsequent mineralization was performed for sample N5 after treatment in an alkaline Ca(OH)_2_ solution. Hardness measurements for sample N5 showed an increase in the HV value in the enamel rods area, compared to both intact enamel (sample N1), the biotemplate without treatment (sample N2), and the alkaline-solution-treated sample (sample N4). The increased value of HV hardness in the area of the enamel rods in sample N5 is apparently associated with changes occurring in the substructure of the mineralized layer, i.e., with the homogeneous crystallization of hydroxyapatite nanocrystals on the biotemplate surface after treatment in alkaline solution and the binding of individual nanocrystals and agglomerates into a single complex by an amino acid booster (Figure 2e and Figure 3e). It should be noted that the HV hardness in the interstitial region in sample N5 is comparable with that in N4 but is lower than that for intact enamel (sample N1). Somewhat lower values of hardness in the interstitial area of sample N5 (as well as N4) are apparently related to the specific substructure of the mineralized layer formed on the biotemplate surface, which according to the AFM data has a uniform micromorphology different from natural enamel. Although the use of alkaline pretreatment of the biotemplate and an amino acid booster led to the formation of a mineralized layer with increased hardness in the areas of the enamel rods, obtaining a mineralizing layer with a similar hierarchy and cleavage characteristics as natural enamel, taking into account the specific micromorphology of dental tissue, is a relevant problem for future research.

Summarizing all the investigations, it should be noted that in previously published research, the effect of some separately utilized amino acids on dental enamel has already been shown [68,69]. We studied the effect of hydroxyapatite mineralization in the presence of amino acids in our previous papers [14,16]. Since polar amino acids play an important role in the protein matrix of enamel, they were applied as an amino acid booster. By varying the amino acid concentrations in the booster, it is possible to obtain a specified morphology of hydroxyapatite [15,17,20]. According to our previous studies, the separately utilized amino acids provide a significant contribution to the crystallization of hydroxyapatite, but their combined effect on hydroxyapatite crystallization under different conditions of biotemplates has not previously been studied. This aspect is of great interest since it opens the possibility of reproducing hierarchical features of enamel at the local level.

Moreover, according to our assumptions, in order to provide ncHAp interaction with the mineralized layer, it was necessary to treat the sample in Ca(OH)_2_ just after the treatment procedure in H_3_PO_4_. The reason for this is a specific interaction of the polar amino acids L-lysine and L-arginine used in our work with ncHAn under different experimental set-up conditions. For example, at low values of pH (acidic medium, pH < 11) in the presence of the polar amino acid L-arginine, formation of hydroxyapatite nanocrystals with a lamellar shape begins [19]. At pH values higher than the isoelectric point (pH > 11), shaft hydroxyapatite crystals are formed in the presence of L-arginine (pH > 11), which are similar to the nanocrystals of apatite in dental enamel [19]. This was the reason for the rejection of an acidic medium when using the amino acid booster. Our investigations also showed [14] that in an acidic medium, a change in the conformational surroundings of the amino acids takes place, as well as a change in the mechanism of their bonding with ncHAp. This results in the failure of the morphological organization required for the reproduction of the specific hierarchical features in dental enamel.

Thus, the choice of the experimental parameters for creating the biomimetic layers, namely, the treatment of the enamel surface with Ca(OH)_2_ and the use of the amino acid booster involving polar amino acids, allowed us to obtain biomimetic mineralized layers (see Figure 3, Figure 5, Figure 6, Figure 11, and Figure 13) with characteristics close to those of the native enamel. Decreasing the remineralization process time will be the topic of further investigations.

## 4. Limitations

In our study, we investigated the formation of a biomimetic mineralizing layer obtained using bioinspired nanocrystalline carbonate-substituted calcium hydroxyapatite on the surface of dental enamel. However, the conditions of the deposition on the biotemplate suitable for clinical applications (the remineralization of enamel) will be different from those described in our work. The biotemplate is in a state different from that of intact enamel. In addition, the small number of samples per class (*n* = 10) is a limitation of the study.

## 5. Conclusions

In this report, we demonstrated the formation of the biomimetic mineralizing layer obtained on the surface of dental enamel (biotemplate) using bioinspired nanocrystalline carbonate-substituted calcium hydroxyapatite (ncHAp), whose physical and chemical properties are closest to the natural apatite dental matrix, together with a complex of polyfunctional organic and polar amino acids. Using a set of structural, spectroscopy and advanced microscopy techniques, we confirmed the formation of a nanosized ncHAp-based mineralized layer and studied its chemical, substructural, and morphological features by means of various methods for the pretreatment of dental enamel. The pretreatment of a biotemplate in an alkaline solution of Ca(OH)_2_ and an amino acid booster, together with the executed subsequent mineralization with ncHAp, led to the formation of a mineralized layer with homogeneous micromorphology and the preferential orientation of the ncHAp nanocrystals. It was shown that homogeneous crystallization of hydroxyapatite on the biotemplate surface and binding of individual nanocrystals and agglomerates into a single complex by an amino acid booster resulted in an increase (~15%) in the nanohardness value in the enamel rods area, compared to that of healthy natural enamel. Obtaining a similar hierarchy and cleavage characteristics as natural enamel in the mineralized layer, taking into account the micromorphological features of dental tissue, is an urgent problem for future research. It will also be important to study how the formation of the layers on the surface of the enamel change under the effects of different diseases (demineralization, caries, and so on).

## Figures and Tables

**Table 1:** Active Raman scattering modes in the spectra of the biomimetic and natural mineralized tissue (enamel), reference samples, and involved materials.

Bond	Wavenumber, cm^−1^	Assignment	References
**Ca–PO_4_^3−^**	139–140	Lattice	[44]
**Ca_II_–OH**	186–191	Lattice	[44]
**Ca–PO_4_^3−^**	201–205	Lattice	[44,51,52]
**Ca–PO_4_^3−^**	231–234	Lattice	[44,51,52]
**Ca–PO_4_^3−^**	263–265	Lattice	[44,51,52]
**Ca_II_–OH**	272–274	Translation	[44,51,52]
**Ca–PO_4_^3−^**	283–285	Libration	[44,51,52]
**Ca_II_–OH**	309–315	Translation	[44,51,52,53]
**Ca_II_–OH**	329–330	Translation	[51,53]
**υ_2_ PO_4_^3−^**	430–432	O-P-O bending, υ_2_	[45,47,48,50,51,54]
**υ_2_ PO_4_^3−^**	445–446	O-P-O bending, υ_2_	[45,47,48,50,51,54]
**υ_4_ PO_4_^3−^**	578–580	O-P-O bending, υ_4_	[45,47,48,50,51,54]
**υ_4_ PO_4_^3−^**	589–591	O-P-O bending, υ_4_	[45,47,48,50,51,54]
**υ_4_ PO_4_^3−^**	608–610	O-P-O bending, υ_4_	[45,47,48,50,51,54]
**υ_4_ PO_4_^3−^**	615–617	O-P-O bending, υ_4_	[45,47,48,50,51,54]
**υ_1_ PO_4_^3−^**	959–962	P-O stretching, enamel, synthetic hydroxyapatite	[45,47,48,50,51,54,55]
**HPO_4_^2−^**	1002–1004	phosphate Sym stretching	[47,56]
**υ_3_ PO_4_^3−^**	1024–1026 (1030 Hap)	P-O asymmetric stretching, enamel, synthetic hydroxyapatite	[47,49,51,57,58,59]
**υ_3_ PO_4_^3−^**	1041–1042	P-O asymmetric stretching	[47,49,51,57,58,59]
**υ_3_ PO_4_^3−^**	1044–1046 (1047 Hap)	P-O asymmetric stretching, enamel, synthetic hydroxyapatite	[47,49,51,57,58,59]
**υ_3_ PO_4_^3−^**	1055–1057	P-O asymmetric stretching	[47,49,51,57,58,59]
**υ_1_ CO_3_ B-type**	1068–1070	PO_4_^3−^ by CO_3_ substitution	[47,49,51,57,58,59]
**υ_3_ PO_4_^3−^**	1075–1078 HAp	P-O asymmetric stretching, enamel, synthetic hydroxyapatite	[47,49,51,57,58,59]
**υ_1_ CO_3_ A-type**	1101–1104	OH by CO_3_ substitution	[47,49,50]

**Table 2:** Characteristic values of R-ratio and Vickers HV hardness of mineralized tissue samples determined from Raman micro-mapping and nanoindentation results for the enamel rod center region (R) and interrod region (IR).

Sample	The Value of the R-Ratio		Vickers HV Hardness	
	IR	R	IR	R
N1	0.64	0.49	418.9 ± 49.7	807.7 ± 95.9
N2	0.69	0.42	490.4 ± 58.2	839.8 ± 99.7
N4	0.56	0.76	498.3 ± 47.3	800.1 ± 95.0
N5	0.55	0.75	373.1 ± 44.1	925.2 ± 109.9

## Data Availability

The data that support the findings of this study are available from the corresponding author upon reasonable request.
